# P299: Assessment of occupational sharps injury reporting and post-exposure management system at King Abdul-Aziz medical city in Jeddah (kamc-j), Saudi Arabia

**DOI:** 10.1186/2047-2994-2-S1-P299

**Published:** 2013-06-20

**Authors:** N Nafouri, B Al Subyei, M Mutasim

**Affiliations:** 1 Infection Prevention & Control, King Abdulaziz medical city - wr, national guard, Jeddah, Saudi Arabia; 2 Infection Prevention & Control, Saudi Arabia; 3 KAMC-WR, Jeddah, Saudi Arabia

## Introduction

Occupational exposure to bloodborne pathogens from sharps injury (SI) is a serious problem, but it is often preventable.

## Objectives

The purpose of this study was to establish a baseline assessment of reporting SI by the staff as well as the efficiency of post-exposure management system.

## Methods

The survey form was adopted from the CDC SI Prevention Workbook but tailored to meet KAMC-J needs. The survey was distributed via department heads.

## Results

A total of 167 healthcare personnel participated from the following work areas: nursing, anesthesia, imaging, dental, oncology, pediatrics and phlebotomy. 159 workers (95%) indicated their knowledge of the reporting protocol and 151 (90%) were familiar with how to manage and report SI. The familiarity of accessing employee injury report (EIR) was reported by 142 (85%) and 145 (87%) specified that they were familiar with how to conduct first aid after exposure. A total of 136 (81%) would report SI to their supervisor first as per the protocol compared to 14 (8%) who would proceed to occupational health and only 11 (7%) would report to emergency room (ER). Only 82 (49%) indicated that they had a previous exposure. 16 workers (20%) followed the protocol by reporting SI then proceeded to receive medical care while 36 (43%) indicated reasons for not reporting an exposure and did not proceed to receive medical care and finally, 30 (37%) indicated reasons for not reporting but received medical care. Getting blamed or to be in trouble for having the exposure was the most ranked reason of not reporting an exposure. The job loss was the least ranked reason. Only 52 (63%) commented on the post exposure experience and 28 workers (54%) indicated that the EIR is a user friendly. Staff health clinic (SHC) was the choice for medical care then ER. SHC worker’s vigilance to report an exposure was the most satisfying quality indicator then the ample time given at the clinic.

## Conclusion

Findings draw attention to the ongoing need to improve reporting communication via periodical educational campaign, surveying staff more often with interventions, utilizing anonymous computer based survey to maximize participation, and simplifying reporting procedure.

## Disclosure of interest

None declared

